# Mosquito diversity (Diptera: Culicidae) and medical importance in four Cambodian forests

**DOI:** 10.1186/s13071-023-05729-w

**Published:** 2023-03-21

**Authors:** Antsa Rakotonirina, Pierre-Olivier Maquart, Claude Flamand, Chea Sokha, Sébastien Boyer

**Affiliations:** 1 Medical and Veterinary Entomology Unit, Institut Pasteur du Cambodge, 983, Phnom Penh, Cambodia; 2 Epidemiology Unit, Institut Pasteur du Cambodge, 983, Phnom Penh, Cambodia; 3 Mathematical Modeling of Infectious Diseases Unit, Institut Pasteur, Université Paris Cité, UMR2000, CNRS, Paris, France; 4 Wildlife Health Program, Wildlife Conservation Society, Phnom Penh, Cambodia; 5 Ecology & Emergence of Arthropod-Borne Pathogens Unit, Department of Global Health, Institut Pasteur, CNRS UMR2000, Paris, France

**Keywords:** Vector mosquitoes, Entomology, Forest, Cambodia

## Abstract

**Background:**

A total of 290 mosquito species are recorded in Cambodia among which 43 are known vectors of pathogens. As Cambodia is heavily affected by deforestation, a potential change in the dynamic of vector-borne diseases (VDBs) could occur through alteration of the diversity and density of sylvatic vector mosquitoes and induce an increase in their interactions with humans. Understanding mosquito diversity is therefore critical, providing valuable data for risk assessments concerning the (re)emergence of local VBDs. Consequently, this study mainly aimed to understand the spatial and temporal distribution of sylvatic mosquito populations of Cambodia by determining which factors impact on their relative abundance and presence.

**Methods:**

A study was conducted in 12 sites from four forests in Cambodia. All mosquitoes, collected during the dry and rainy seasons, were morphologically identified. The diversity and relative density of mosquito species in each site were calculated along with the influence of meteorological and geographical factors using a quasi-Poisson generalized linear model.

**Results:**

A total of 9392 mosquitoes were collected belonging to 13 genera and 85 species. The most represented genera were *Culex*, accounting for 46% of collected mosquitoes, and *Aedes* (42%). Besides being the most abundant species, *Culex pseudovishnui* and *Aedes albopictus*, which are known vectors of numerous arboviruses, were present in all sites during both dry and rainy seasons. The presence of mosquito species reported to be zoo-anthropophilic feeders was also observed in both forested and urban areas. Finally, this study demonstrated that altitude, temperature and precipitation impacted the abundance of mosquitoes but also influenced species community composition.

**Conclusion:**

The results indicate an important diversity of mosquitoes in the four forests and an influence of meteorological and geographical factors on their community. Additionally, this work highlights in parallel the abundance of species considered to be of medical importance and therefore underlines the high risk of pathogen emergence/re-emergence in the region.

**Graphical Abstract:**

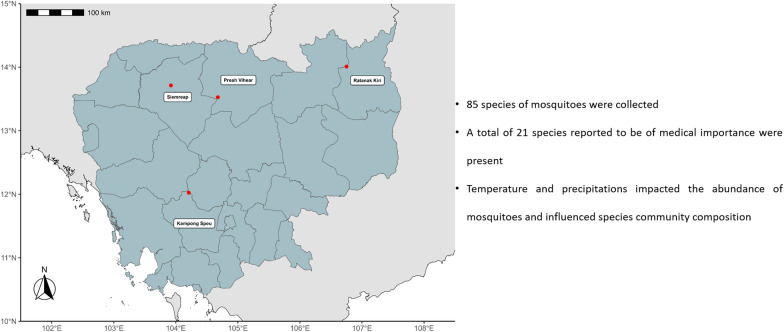

**Supplementary Information:**

The online version contains supplementary material available at 10.1186/s13071-023-05729-w.

## Background

Vector-borne diseases (VBDs) are a major public health problem worldwide. In 2020, the World Health Organization estimated that they account for > 17% of all infectious diseases worldwide and are responsible for > 700,000 deaths per year, overburdening health systems mainly in the tropical and subtropical areas [1, 2].

Cambodia is affected by VBDs where dengue fever and Japanese encephalitis (JE) are endemic [3, 4]. Specifically, Cambodia has one of the highest dengue infection rates in Southeast Asia, with an average of 103 cases per 10,000 population and a case fatality rate of 1 to 2% since 2000 [5]. Beyond the public health issue it represents, dengue fever is also responsible for a heavy societal burden in Cambodia with a significant cost of illness [6]. JE is the main cause of central nervous system infections leading to encephalitis and other serious clinical complications in Cambodian children [4]. In 2007, the estimated incidence of clinically reported JE in the country was 11.1 cases per 100,000 children < 15 years of age [4]. A recent resurgence of chikungunya was also recorded in the country in 2011, breaking out in the village of Trapeang Roka (Kampong Speu Province) in 2012 [7]; later, in 2020, a nationwide outbreak occurred [8]. Additionally, silent circulation of Zika fever was confirmed in Cambodia [9], and malaria still occurs, accounting for 13.4% of cases in the Southeast Asia region in 2020 [10]. These VBDs are caused by pathogens, namely dengue, Japanese encephalitis, chikungunya and Zika viruses (DENV, JEV, CHIKV and ZIKV, respectively) and *Plasmodium*, which are transmitted to humans through the bite of vector mosquitoes. To date, 43 confirmed vector species of pathogens have been recorded in Cambodia [11].

Land use change, such as deforestation and urbanization, heavily affects Cambodia: the country lost 65% of its forest coverage from 2006 to 2016 [12–14]. This alteration could modify the dynamic of VBDs by potentially changing mosquito communities and abundance. Indeed, several meta-analyses, combining data from different countries, have highlighted that mosquito species can be affected by deforestation, in some cases leading to an increase in their abundance, especially for species associated with VBDs [15, 16]. This potential increase in the abundance of vector mosquitoes directly impacts their vectorial capacity (i.e. the efficiency of the transmission in a specific vector-host relationship in a given environment [17]) and could be multifactorial. This may result from the creation of breeding habitats more favorable for the immature stages [18] or the enhancement of mosquito survival and reproduction due to deforestation-induced microclimate modification [19, 20]. Moreover, deforestation can also result in increased human interaction with wildlife [21] and consequently the likelihood of human-vector contact and (re)emergence of pathogens [22].

In this context, describing the mosquito diversity and relative abundance in Cambodian forests is essential for VBD risk assessments and public health recommendations. Different works have already explored the mosquito fauna in forested areas of Cambodia. Recent studies conducted in the bird sanctuary in Prek Toal flooded forest in Battambang Province and the mangrove forest in Koh Kong have overviewed the overall Culicidae fauna [22, 23]; other works focused only on *Anopheles* mosquitoes. The first study of *Anopheles* in Cambodia dates back to 1964 in two villages and the surrounded forests of Pailin Province [24]. Other studies have provided insights into the Anophelinae fauna in different forests or villages inside the forests (or surrounded by forests), sometimes through vector control studies or the evaluation of *Anopheles* capture methods [25–30]. The sites surveyed during these studies were located in Mondulkiri, Pailin, Preah Vihear, Pursat and Ratanak Kiri Provinces.

The extension of these studies to other forests in Cambodia and to the entire Culicidae fauna is strongly recommended to better characterize sylvatic mosquito species. Therefore, the main objective of this work was to examine the spatio-temporal distribution of mosquitoes in Cambodian forests including species vectors of pathogens. The secondary objective was to determine the meteorological and geographical variables that could explain their relative abundance and presence.

## Methods

### Study sites

**Fig. 1:**
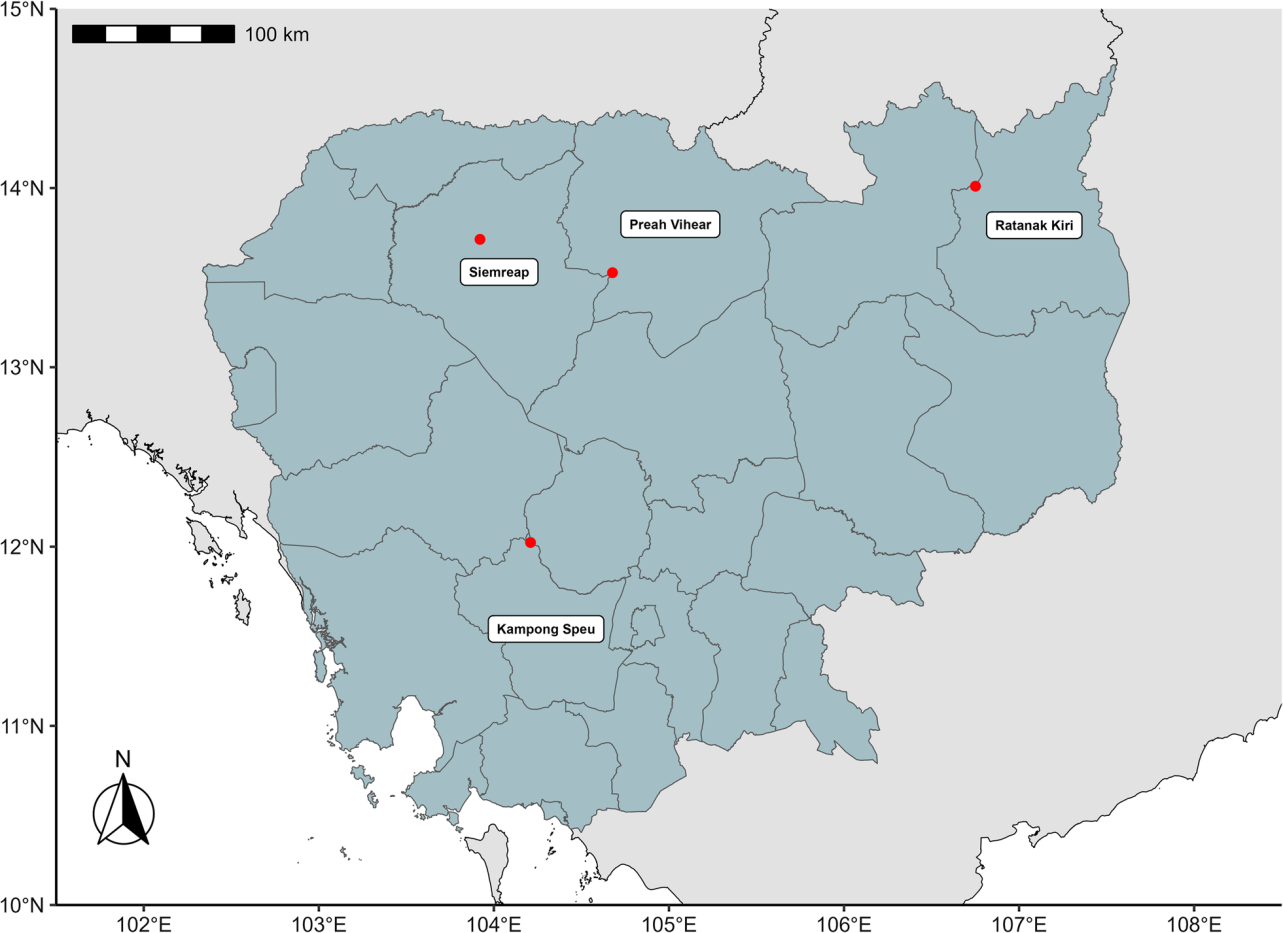
Map of Cambodia indicating the study areas in Kampong Speu, Preah Vihear, Ratanak Kiri and Siemreap. The red points indicate the sites. The map was created with R studio software

The study was conducted in four different forests located in Kampong Speu, Preah Vihear, Ratanak Kiri and Siemreap (Fig. 1).

Sampling forests were selected to represent different protected areas in the north, northeast and south of Cambodia. These were the forests for which approval from the Cambodian authorities was obtained. Three sites per forest were surveyed corresponding respectively to the depth, middle and edge of the forest. The description of each site is presented in Table 1.

**Table 1:** Description of the different sites

Forests	Sites	Localization		Average of temperature in 2020 (°C)	Annual precipitation in 2020 (mm)	Altitude range (m)	Characteristics
Kampong Speu	1	104.1432°E, 11.5811°N		26.7	1944.52	300–401	Evergreen forest: Presence of many bamboo trees
	2	104.1416E, 11.5743N		25.4	1944.52	300–316	Evergreen forest: Presence of many bamboo trees Presence of stream
	3	104.1758E, 11.5201N		24.9	1903.48	122	Rural village
Preah Vihear	1	104.4246E, 13.2936N		27.4	1760.13	125–194	Semi-evergreen forests
	2	104.4338E, 13.2940N		27.4	1760.13	82–94	
	3	104.7374E, 13.4933N		26.4	1934.32	70–72	
Ratanak Kiri	1	106.4451E, 14.1367N		26.1	2572.77	112–130	Semi-evergreen forest: Dry-savannah ecosystem on forest edges
	2	106.4454E, 14.1283N		26.1	2572.77	122–128	
	3	106.4454E, 14.1812N		26.1	2649.71	113–115	Ranger station
Siemreap	1	103.5452E, 13.4445N	26.8		1436.06	102–122	Semi-evergreen forest: Presence of zoo Presence of bat cave
	2	103.5259E, 13.4630N	26.9		1402.63	150–162	Semi-evergreen forest
	3	104.1327E, 13.4404N	27.9		1681.07	75–107	Ranger station

### Mosquito sampling and morphological identification

Mosquito sampling in these selected sites was carried out between March 2020 and January 2021. Two field missions were conducted in each forest, one during the dry season and one during the rainy season (with the exception of Kampong Speu where the two missions were conducted during the rainy season for logistical reasons).

Two types of traps were used to collect adult mosquitoes: BG-1 Sentinel™ Mosquito Traps, 7.5–12 volts baited with BG-Lure® (BioQuip, Rancho Dominguez, CA, USA) and CDC Mini Light Traps (BioQuip) with incandescent light. Dry ice was placed in a dry ice dispenser next to each trap. For each mission, these traps were set for 3 consecutive days per site and harvested every 24 h.

Collected mosquitoes were subsequently killed humanely using carbon dioxide. These were morphologically identified by using available identification keys [31–34].

### Meteorological and geographical data

**Fig. 2:**
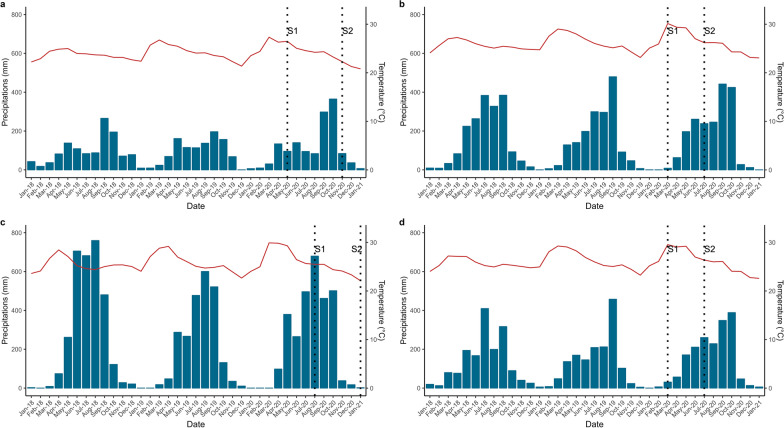
Meteorological conditions in the four forests during the year of collection and 2 years before (2018, 2019 and 2020). **a** Meteorological conditions in Kampong Speu Forest. **b** Meteorological conditions in Ratanak Kiri Forest. **c** Meteorological conditions in Siemreap Forest. **d** Meteorological conditions in Siemreap Forest. Temperatures (red line) were gathered from CFSR satellite data (19.2 km/28.28 km, daily) and precipitation (blue bars) from CHIRPS satellite data (4.8 km, daily)

Meteorological data were obtained from app.climateengine.com (accessed on 15 June 2022). The temperature was extracted from CFSR satellite data (19.2 km/28.28 km, daily) and precipitation from CHIRPS satellite data (4.8 km, daily). The meteorological conditions (that could impact the mosquito community) during the year of collection did not differ from those of the previous years (Fig. 2). Moreover, the altitude values at each global positioning system (GPS) data point were obtained directly with Google Earth Pro (version 7.3.6.9345).

### Data analysis

All the data analyses were performed using R software [35]. First, to assess the composition of the mosquito community in each site, three indices were computed: Shannon’s diversity index (H′) quantifying the species diversity, Simpson index (D) measuring the species dominance and Pielou’s evenness index (Jʹ) calculating whether species are distributed evenly. The equations of these indices are shown here:

#### Shannon diversity index

$$H^{\prime} = - \sum {\left( \frac{ni}{N} \right){*}\log \left( \frac{ni}{N} \right)}$$

where “ni” is the number of specimens belonging to one species and “N” is the total number of specimens from all species in the site.

#### Simpson index

$${\text{D}} = 1 - \sum {Pi^{2} }$$

where Pi is the proportion of specimens belonging to a species and calculated by dividing “ni” by “N.”

#### Pielou’s evenness index

$$J^{\prime} = \frac{{H^{\prime}}}{{H^{\prime} \max }}$$

where “H” is the Shannon diversity index and “*H*’max” the maximum possible value of *H*’ if every species is equally likely.

A non-parametric Wilcoxon test was carried out to compare the relative abundance of mosquitoes during the dry and rainy seasons. Then, the correlation between the relative abundance of mosquito species in the different sites was also computed with Pearson tests. Only the species whose number was ≥ 5 was included in the analysis.

Finally, the relationship between mosquito relative abundance and geographical and meteorological factors was evaluated. The meteorological factors (i.e. temperature and precipitations) were classified with a time lag of 1 to 4 weeks prior to collection. One of the distributions commonly used to model count data is the Poisson distribution. However, due to a significant overdispersion of the residuals of the Poisson model, a quasi-Poisson generalized linear model was applied. The collinearity between the different variables was also tested to avoid combining highly correlated variables. An abundance model was performed for all mosquito species whose number was ≥ 40 while a presence model was carried out for mosquito species whose number was < 40. The relative risks (RRs) and 95% confidence intervals (IC_95_) were calculated to quantify the influence of these factors on relative abundance. The statistical significance threshold for all tests was set at 0.05.

## Results

### Mosquito diversity and relative abundance

#### Overall results

A total of 9392 mosquitoes were collected representing 85 species belonging to 13 genera (Table 2). The genera collected were *Aedes* (17 species)*, Culex* (16 species), *Uranotaenia* (13 species), *Anopheles* (11 species), *Armigeres* (10 species), *Heizmannia* (5 species), *Mansonia, Mimomyia* and *Tripteroides* (3 species each), and *Aedeomyia, Coquillettidia, Lutzia* and *Toxorhynchites* (1 species each).

**Table 2:** Overview of collected mosquitoes per forest and per season

Genus	Species	Kampong Speu		Preah Vihear		Ratanak Kiri		Siemreap		Total	Genus (%)	Species (%)	*N* forests
		Rainy1	Rainy2	Dry	Rainy	Dry	Rainy	Dry	Rainy				
*Aedeomyia*	*Ad. catasticta*	0	0	0	0	1	0	0	0	1	0.01	0.01	1
*Aedes*	Ae. aegypti^*^	20	0	0	0	0	0	0	0	20	42.01	0.21	1
	*Ae. albolineatus*	0	170	0	0	0	0	0	0	170		1.81	1
	Ae. albopictus^*^	247	238	6	226	123	85	82	387	1394		14.84	4
	*Ae. annulirostris*	0	0	0	2	0	0	0	0	2		0.02	1
	*Ae. caecus*	0	0	0	0	1	0	0	0	1		0.01	1
	*Ae. desmotes*	65	62	0	0	0	0	0	0	127		1.35	1
	*Ae. eldridgei*	0	47	0	0	0	0	0	0	47		0.50	1
	*Ae. elsiae*	0	2	0	0	0	0	0	0	2		0.02	1
	*Ae. feegradei*	0	0	0	0	0	3	0	0	3		0.03	1
	*Ae. gardneri imitator*	1	5	0	64	8	40	0	284	402		4.28	4
	*Ae. ibis*	0	1	0	2	2	6	0	3	14		0.15	4
	*Ae. imprimens*	0	0	0	0	1	0	0	1	2		0.02	2
	*Ae. ostentatio*	0	0	0	0	0	8	0	0	8		0.09	1
	*Ae. prominens*	0	1	0	0	0	1	0	6	8		0.09	3
	*Ae. thailandensis*	0	0	0	0	0	1	0	0	1		0.01	1
	Ae. vexans^*^	0	0	0	4	0	0	0	4	8		0.09	2
	*Ae. vittatus*	1	2	0	0	0	0	0	1	4		0.04	2
	*Ae.* sp.	877	259	2	146	55	77	4	313	1733		18.45	4
*Anopheles*	*An. baimaii*	1	2	0	0	0	0	0	0	3	0.70	0.03	1
	An. barbirostris^*^	0	0	0	0	1	0	0	0	1		0.01	1
	An. campestris^*^	4	0	0	0	0	0	0	0	4		0.04	1
	*An. interruptus*	0	0	0	0	1	1	0	0	2		0.02	1
	An. karwari^*^	0	0	0	0	0	2	0	0	2		0.02	1
	An. maculatus^*^	0	0	1	0	0	0	0	3	4		0.04	2
	An. minimus^*^	0	0	0	0	1	0	0	0	1		0.01	1
	An. nivipes^*^	0	2	0	1	0	0	0	0	3		0.03	2
	*An. peditaeniatus*	0	0	0	0	0	0	1	0	1		0.01	1
	An. philippinensis^*^	0	0	0	1	0	0	0	0	1		0.01	1
	*An. umbrosus*	0	0	0	0	0	1	0	0	1		0.01	1
	*An.* sp.	5	13	6	7	7	1	0	4	43		0.46	4
*Armigeres*	*Ar. annulitarsis*	1	36	0	0	1	4	0	0	42	3.49	0.45	2
	*Ar. aureolineatus*	0	0	0	0	0	1	0	0	1		0.01	1
	*Ar. dolichocephalus*	0	0	0	0	3	0	0	0	3		0.03	1
	*Ar. flavus*	0	1	0	0	0	0	0	0	1		0.01	1
	*Ar. kesseli*	0	0	0	0	0	1	0	8	9		0.10	2
	*Ar. longipalpis*	0	2	0	0	0	0	0	0	2		0.02	1
	*Ar. malayi*	0	2	0	0	0	0	0	0	2		0.02	1
	*Ar. omissus*	2	1	0	0	0	0	0	0	3		0.03	1
	Ar. subalbatus*	3	7	3	6	72	71	1	16	179		1.91	4
	*Ar. theobaldi*	0	1	0	0	0	0	0	0	1		0.01	1
	*Ar.* sp.	16	32	0	2	4	24	0	7	85		0.91	4
*Coquillettidia*	Cq. crassipes*	10	17	4	1	2	1	2	5	42	0.45	0.45	4
*Culex*	Cx. bitaeniorhynchus*	0	70	1	4	2	5	0	5	87	45.58	0.93	4
	*Cx. brevipalpis*	0	10	0	7	0	2	13	1008	1040		11.07	4
	*Cx. cinctellus*	0	227	0	0	1	0	4	0	232		2.47	3
	*Cx. fraudatrix*	0	52	0	0	10	0	0	0	62		0.66	2
	Cx. fuscocephala*	1	4	0	0	0	2	1	0	8		0.09	3
	Cx. gelidus*	0	0	0	0	0	0	2	4	6		0.06	1
	*Cx. infantulus*	0	0	0	0	0	0	1	0	1		0.01	1
	*Cx. macdonaldi*	0	0	0	0	0	0	0	2	2		0.02	1
	*Cx. mimulus*	5	1	0	0	3	0	0	3	12		0.13	3
	*Cx. nigropunctatus*	4	38	1	35	1	5	2	40	126		1.34	4
	*Cx. perplexus*	0	0	0	0	0	1	0	1	2		0.02	2
	Cx. quinquefasciatus*	16	5	0	0	0	0	0	8	29		0.31	2
	*Cx. sinensis*	1	1	0	2	0	2	0	0	6		0.06	3
	Cx. sitiens*	0	3	0	0	0	3	0	0	6		0.06	2
	Cx. pseudovishnui*	99	113	25	72	416	1177	395	61	2358		25.11	4
	*Cx. whitmorei*	0	2	0	0	0	0	0	0	2		0.02	1
	*Cx.* sp.	22	108	21	12	57	11	9	62	302		3.22	4
*Heizmannia*	*Hz. catesi*	0	0	0	0	0	0	0	1	1	2.40	0.01	1
	*Hz. chengi*	0	0	0	0	0	3	0	3	6		0.06	2
	*Hz. complex*	0	0	0	0	4	0	0	1	5		0.05	2
	*Hz. demeilloni*	0	0	0	4	5	43	0	0	52		0.55	2
	*Hz. reidi*	0	0	0	0	0	1	0	0	1		0.01	1
	*Hz*. sp.	5	34	0	18	28	58	1	16	160		1.70	4
*Lutzia*	*Lutzia vorax*	0	0	0	0	0	0	0	1	1	0.03	0.01	1
	*Lutzia* sp.	0	1	1	0	0	0	0	0	2		0.02	2
*Mansonia*	Ma. annulifera*	0	0	0	0	0	0	0	3	3	0.22	0.03	1
	Ma. indiana*	0	0	0	0	0	1	0	4	5		0.05	2
	Ma. uniformis*	2	0	0	0	0	0	6	2	10		0.11	2
	*Ma.* sp.	2	0	0	0	0	0	0	1	3		0.03	2
*Mimomyia*	*Mi. aurea*	0	1	0	0	0	0	0	0	1	0.18	0.01	1
	*Mi. hybrida*	0	0	0	0	0	0	0	9	9		0.10	1
	*Mi. luzonensis*	0	3	0	0	0	0	0	0	3		0.03	1
	*Mi.* sp.	1	1	1	0	0	0	0	1	4		0.04	3
*Tripteroides*	*Tr. aranoides*	0	2	0	0	0	0	0	0	2	0.28	0.02	1
	*Tr. caeruleocephalus*	0	1	0	0	0	0	0	0	1		0.01	1
	*Tr. powelli*	0	0	0	0	0	0	2	0	2		0.02	1
	*Tr.* sp.	3	14	4	0	0	0	0	0	21		0.22	2
*Toxorhynchites*	*Tx. splendens*	0	0	0	5	0	0	0	0	5	0.09	0.05	1
	*Tx.* sp.	0	0	0	1	0	1	0	1	3		0.03	3
*Uranotaenia*	*Ur. bicolor*	0	0	0	0	5	0	0	1	6	4.45	0.06	2
	*Ur. bimaculiala*	0	0	0	0	0	0	2	3	5		0.05	1
	*Ur. campestris*	0	0	0	0	0	0	1	0	1		0.01	1
	*Ur. koli*	4	14	0	0	1	0	0	0	19		0.20	2
	*Ur. longirostris*	4	0	0	0	0	0	1	17	22		0.23	2
	*Ur. lutescens*	0	3	0	0	0	0	0	0	3		0.03	1
	*Ur. macfarlanei*	0	17	0	0	66	0	0	0	83		0.88	2
	*Ur. maxima*	0	0	0	0	1	0	0	0	1		0.01	1
	*Ur. metatarsata*	0	1	0	0	0	0	5	0	6		0.06	2
	*Ur. micans*	0	3	0	0	0	0	0	0	3		0.03	1
	*Ur. rampae*	0	1	0	0	0	1	0	0	2		0.02	2
	*Ur. testacea*	0	5	0	0	0	0	0	0	5		0.05	1
	*Ur. trilineata*	0	0	0	0	0	0	0	1	1		0.01	1
	*Ur.* sp.	5	64	1	9	153	9	6	14	261		2.78	4
Unidentified		0	1	0	1	1	4	0	3	10	0.11	0.11	5
Overall total		3130		709		2496		2859		9392	**–**	**–**	**–**
Overall genera		12		11		11		10		13	**–**	**–**	**–**
Overall species		46		17		42		39		85	**–**	**–**	**–**
Total per forest per season		1427	1703	77	632	1037	–	–	–	9392	**-**	**-**	**-**
Number of genera per forest per season		10	10	9	8	8	–	–	–	13	**-**	**-**	**-**
Number of species per forest per season		22	44	12	17	24	–	–	–	85	**-**	**-**	**-**
Shannon index (H’)		1.61	2.51	1.36	1.54	1.46	–	–	–	-	**-**	**-**	**-**
Simpson index (D)		0.31	0.12	0.39	0.32	0.37	–	–	–	-	**-**	**-**	**-**
Pielou’s evenness index (J’)		0.23	0.23	0.29	0.24	0.20	0.11	0.14	0.19				

N sites: number of sites where the species was present

^*^Species vectors of pathogens [11]

The *Culex* genus was the most abundant one, accounting for 45.58% (*n* = 4281) of the total collected mosquitoes, followed by *Aedes* with 42.01% (*n* = 3946) of our collection. The third most abundant genus was *Uranotaenia* accounting for 4.45% (*n* = 418), while *Armigeres* and *Heizmannia* represented 3.49% (*n* = 328) and 2.40% (*n* = 225), respectively. In our study, the genus *Anopheles* represented only 0.7% (*n* = 66) of the collected specimens.

Overall, two dominant species were observed: *Culex pseudovishnui*, accounting for 25.11% (*n* = 2358) of mosquitoes, followed by *Ae. albopictus* (*n* = 1394; 14.84%). A total of 21 species are reported to be of medical importance (*n* = 4132; 43.99%).

#### Results per forest and per site

With a total of 3130 mosquitoes belonging to 46 species, the Kampong Speu Forest displayed the largest diversity of mosquitoes. In contrast, the forest in Preah Vihear had only a total of 709 mosquitoes belonging to 17 species (Table 2).

Nine mosquito species were common in the four forests, namely (ranked by abundance) *Cx. pseudovishnui*, *Ae. albopictus, Culex brevipalpis, Aedes gardneri imitator, Armigeres subalbatus, Cx. nigropunctatus, Cx. bitaeniorhynchus, Coquillettidia crassipes* and *Ae. ibis* (Table 2). Two of them, *Ae.* *albopictus* and *Cx.* *pseudovishnuii*, were present in all sites (Additional file 1: Table S1) regardless of the season (Table 2). Two species, *Ar. subalbatus* and *Ae. gardneri imitator*, were collected in 11 of the 12 sites in the four forests independently of the season (Additional file 1: Table S1).

In addition, 12 other species common to the forested and anthropized areas (rural village or ranger station) were found. These were *Aedes albolineatus, Ae. desmotes, Ae. gardneri imitator, Armigeres annulitaris, Ar. kesseli, Ar. subalbatus*, *Cq. crassipes, Culex bitaeniorhynchus, Cx. brevipalpis, Cx. nigropunctatus, Cx. quinquefasciatus* and *Uranotaenia* *macfarlanei* (Additional file 1: Table S1). Thirty-nine other species were only found at a single site of a single forest (Additional file 1: Table S1).

**Fig. 3:**
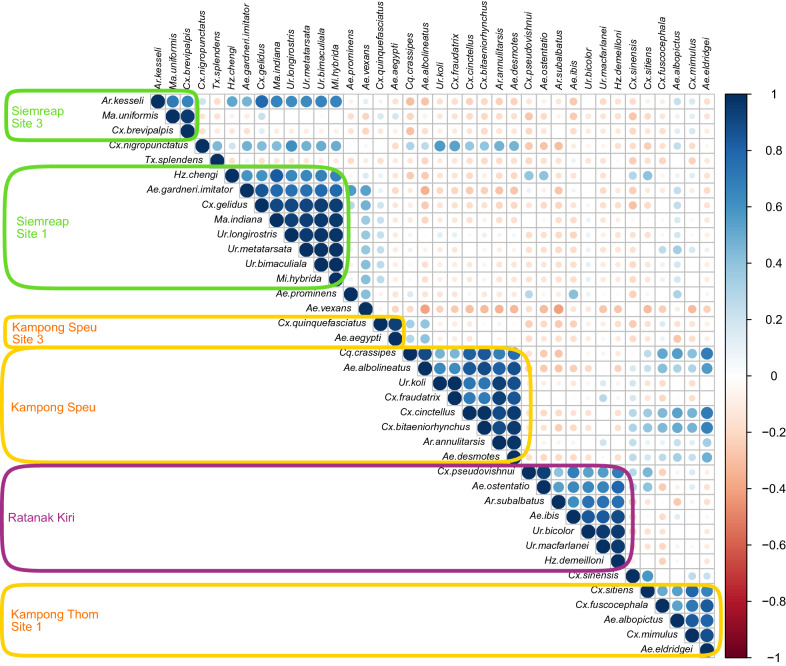
Correlation matrix representing Pearson correlation between the relative abundance of species in each site. The size of the circle and color intensity are relative to the correlation coefficients (the values of correlations coefficient are presented in the Additional file 2: Table S2). Negative correlations are shown in red and positive correlations in blue. On the right, the legend shows the corresponding colors and the correlation coefficients. The different boxes represent the different study sites

Also, a strong positive correlation between the relative abundance of different species due to their co-occurrence in the same site was observed (Additional files 2 and 3: Tables S2 and S3, Fig. 3). This was the case for *Mansonia uniformis* and *Cx. brevipalpis* mostly collected in site 3 of Siemreap Forest but also for *Ae. gardneri imitator, Culex gelidus, Mansonia indiana, Uranotaenia longirostris, Ur. metatarsata, Ur. bimaculiala* and *Mimomyia hybrida* mostly found in site 1 of Siemreap Forest. *Aedes aegypti* and *Cx. quinquefasciatus* were mainly collected in site 3 of Kampong Speu Forest while *Ae. albolineatus* and *Cq. crassipes* were mainly present across the three sites of Kampong Speu. Six other species, *Ae. desmotes, Ar. annulitarsis, Culex bitaeniorhynchus, Cx. cinctellus, Cx. fraudatrix* and *Uranotaenia koli*, were mostly found in sites 1 and/or 2 of the Kampong Speu Forest. *Aedes ibis, Ae. ostentatio, Ar. subalbatus, Cx. pseudovishnui, Heizmanni demeilloni, Uranotaenia bicolor* and *Ur. macfarlanei* were mainly present in sites 1 and/or 2 of Ratanak Kiri Forest. Finally, *Aedes eldridgei* and *Culex mimulus* were mostly collected in site 1 of Kampong Speu Forest.

### Seasonal relative abundance and diversity of mosquitoes

The relative abundance of Culicidae increased significantly during the rainy season compared to the dry season in Preah Vihear (Wilcoxon test, *P* = 0.001) and Siemreap (Wilcoxon test, *P* = 2.5 × 10^–05^). Moreover, the number of mosquito species also increased significantly in these two forests during the rainy season. It went from 12 to 17 species in Preah Vihear (Wilcoxon test, *P* = 0.007) and from 18 to 33 in Siemreap Forest (Wilcoxon test, *P* = 0.005).

A change in Shannon, Simpson and Pielou’s evenness indices between the dry and rainy seasons was highlighted. A decrease of Shannon index was observed during the dry season in Preah Vihear and Siemreap (Table 2). In contrast, in Ratanak Kiri, a decrease of Shannon and Pielou’s indices was observed during the rainy season with a dominance of *Cx.* *pseudovishnui*.

### Impact of meteorological and geographical factors on the relative abundance of mosquito species

Altitude, ranging from 75 to 401 m above sea level, showed mainly a slight positive impact on the relative abundance and presence of mosquito species (Table 3 and Additional file 4: Table S4). Specifically, a positive correlation between altitude and the abundance of five *Aedes* species (*Ae. albolineatus, Ae. albopictus, Ae. desmotes, Ae. eldridgei, Ae. gardneri*) was found as well as the abundance of *Cq. crassipes* and three *Culex* species (*Cx. bitaeniorhynchus, Cx. cinctellus*, *Cx. fraudatrix*). In contrast, a negative correlation between the altitude and the relative abundance of *Ar. subalbatus* and*Cx. brevipalpis* was observed. Moreover, the presence of *Ma. uniformis* was negatively impacted by altitude while this factor impacted positively on the presence of *Ur. koli* (Additional file 4: Table S4).

**Table 3:** Result of regression model showing the correlation between the relative abundance of species and meteorological/geographical variables

Factors	Species	IRR	IC_95_ inf	IC_95_ sup	*P*-value
Altitude	*Ae. albolineatus* ^(+)^	1.01	1.00	1.01	4 × 10^–5^
	*Ae. albopictus* ^(+)^	1.00	1.00	1.01	3 × 10^–4^
	*Ae. desmotes* ^(+)^	1.01	1.01	1.02	10^–4^
	*Ae. eldridgei* ^(+)^	1.01	1.01	1.02	2 × 10^–16^
	*Ae. gardneri* ^(+)^	1.00	0.99	1.00	0.04
	*Ar. annulitarsis* ^(+)^	1.01	1.01	1.01	10^–15^
	*Ar. subalbatus* ^(−)^	0.99	0.98	1.00	0.02
	*Cq. crassipes* ^(+)^	1.01	1.00	1.01	4 × 10^–3^
	*Cx. bitaeniorhynchus* ^(+)^	1.01	1.00	1.01	2 × 10^–7^
	*Cx. brevipalpis* ^(−)^	0.99	0.89	1.02	10^–3^
	*Cx. cinctellus* ^(+)^	1.01	1.01	1.02	2 × 10^–16^
	*Cx. fraudatrix* ^(+)^	1.05	1.03	1.08	3 × 10^–6^
	*Cx. nigropunctatus*	1.00	1.00	1.01	0.22
	*Cx. pseudovishnui*	1.00	0.99	1.00	0.12
	*Hz. demeilloni*	0.98	0.95	1.00	0.08
	*Ur. macfarlnei*	1.01	1.00	1.01	0.62
Precipitation 1st week before the collection	*Ae. desmotes*	1.05	0.87	1.20	0.55
	*Hz. demeilloni* ^(+)^	1.25	1.14	1.44	3 × 10^–7^
Precipitation 2nd week before the collection	*Ae. albolineatus* ^(+)^	1.35	1.17	1.61	3 × 10^–6^
	*Ae. gardneri* ^(+)^	1.14	1.05	1.26	2 × 10^–3^
	*Ar. annulitarsis* ^(+)^	1.22	1.15	1.32	3 × 10^–11^
	*Cx. bitaeniorhynchus* ^(+)^	1.14	1.07	1.23	6 × 10^–5^
	*Cx. brevipalpis*	1.08	0.71	1.47	0.67
Precipitation 3rd week before the collection	*Ae. eldridgei* ^(+)^	1.53	1.29	1.86	10^–10^
	*Ar. subalbatus* ^(+)^	1.06	1.02	1.10	2 × 10^–03^
	*Cq. crassipes*	0.99	0.93	1.04	0.62
	*Cx. cinctellus*	0.98	0.92	1.04	0.56
	*Cx. fraudatrix* ^(−)^	0.47	0.24	0.64	10^–13^
	*Cx. nigropunctatus*	0.97	0.92	1.02	0.22
	*Cx. pseudovishnui* ^(+)^	1.06	1.04	1.09	3 × 10^–07^
	*Ur. macfarlnei*	0.85	0.65	1.02	0.08
Precipitation 4rd week before the collection	*Ae. albopictus*	1.02	0.99	1.06	0.11
Temperature 1st week before collection	*Ae. eldridgei* ^(−)^	0.08	0.02	0.26	4 × 10^–09^
	*Ar. annulitarsis* ^(−)^	0.60	0.43	0.81	4 × 10^–07^
	*Cx. bitaeniorhynchus* ^(−)^	0.69	0.52	0.88	3 × 10^–04^
Temperature 2nd week before collection	*Ar. subalbatus* ^(−)^	0.64	0.48	0.81	4 × 10^–04^
	*Cq. crassipes*	1.07	0.87	1.34	0.40
	*Cx. fraudatrix* ^(−)^	0.11	0.01	0.27	4 × 10^–07^
	*Cx. pseudovishnui*	0.90	0.75	1.08	0.15
Temperature 3rd week before collection	*Ae. albolineatus* ^(−)^	0.16	0.05	0.44	2 × 10^–04^
	*Cx. brevipalpis* ^(+)^	8.26	1.37	2182.44	4 × 10^–03^
	*Cx. cinctellus* ^(−)^	0.58	0.44	0.74	2 × 10^–07^
	*Cx. nigropunctatus*	1.11	0.95	1.32	0.08
	*Hz. demeilloni*	0.50	0.24	0.83	0.10
Temperature 4th week before collection	*Ae. albopictus* ^(+)^	1.40	1.22	1.63	10^–08^
	*Ae. desmotes*	1.23	0.83	1.88	0.35
	*Ae. gardneri* ^(+)^	2.07	1.47	3.04	8 × 10^–05^
	*Ur. macfarlnei*	0.33	0.19	0.49	0.62

ANOVA test. ( +): positive correlation, (−): negative correlation

*IRR* Incidence Ratio Rate

The study also demonstrated that the precipitation impacted the relative abundance and presence of mosquitoes mainly positively (Table 3 and Additional file 4: Table S4). The average precipitation in the first week prior to the collection impacted the abundance of *Hz. demeilloni* positively and the presence of *Ur. longirostris* negatively. The average precipitation in the second week prior to the sampling also impacted the relative abundance of *Ae. albolineatus, Ae. gardneri, Ar. annulitarsis* and *Cx. bitaeniorhynchus* and the presence of *Ur. koli* positively. The average precipitation in the third week before the mosquito collection impacted the relative abundance of *Ae. eldridgei, Ar. subalbatus* and *Cx. pseudovishnui* and the presence of *Ae. ibis* positively as well. The average precipitation in the third week prior to the collection had a significant negative impact on the relative abundance of *Cx. fraudatrix*. Similarly, the average precipitation in the fourth week before the collection impacted the presence of *Ae. aegypti* negatively.

In contrast, our results demonstrated that for all the time lags, the temperature mainly impacted the relative abundance and presence of mosquitoes negatively (Table 3 and Additional file 4: Table S4). The average temperature in the first week before the sampling impacted the abundance of *Ae. eldridgei, Ar. annulitarsis* and *Cx. bitaeniorhynchus* negatively. The average temperature during the second week before the collection impacted the abundance of *Ar. subalbatus* and *Cx. fraudatrix* negatively and the presence of *Ae. aegypti* positively. The temperature in the third week before the collection impacted the relative abundance of *Ae. albolineatus* and *Cx. cinctellus* negatively and the abundance of *Cx. brevipalpis* positively. Finally, the temperature in the fourth week before the collection had a significant positive impact on the relative abundance of *Ae. albopictus* and *Ae. gardneri* and on the presence of *Ma. uniformis* and *Ur. longirostris*.

## Discussion

The overall mosquito fauna in the four forests was quite diverse but the relative abundance showed a dominance of *Culex* mosquitoes. The same result has been observed in other Cambodian forests [22, 23] and also in different urban, peri-urban and rural areas of Cambodia [36–38]. However, the dominant *Culex* species changed according to the biotope. In our study, *Cx. pseudovishnui* was mainly the dominant species regardless of the type of forest. Little is known about the biology of *Cx. pseudovishnui* [33, 39, 40]. During a previous study, it was found to be more abundant during the rainy season [40], which was confirmed by our study, except in Siemreap Forest where this species was surprisingly more abundant during the dry season. *Culex cinctellus* was the most abundant *Culex* species in the bamboo forests of Kampong Speu where the traps were set next to a stream. Interestingly, at this site, this species was only collected in November. These observations confirm the previously described breeding habitat and seasonality of *Cx.* *cinctellus* reported in Thailand, where mosquitoes have been collected in a bamboo forest on October and November [39]. *Culex quinquefasciatus* was the most dominant *Culex* species in the rural village of Kampong Speu; it is a common species in rural and urban areas [41, 42]. Moreover, *Cx. brevipalpis* was the dominant *Culex* species in the zoo of Siemreap Forest. According to the literature, this species is able to colonize different breeding habitats, and humans are not the usual hosts [33, 39].

*Aedes* was the second most abundant mosquitoe in our study. This genus is the second most diversified in Cambodia [11] but its relative abundance has been found to be less important in other forests [22, 23] and in anthropized areas of Cambodia [36–38]. *Aedes albopictus* was the most abundant *Aedes* species in this work and was present in all the sites*.* Its presence and abundance could be explained by its sylvatic origin in the tropical forest areas of Southeast Asia [43] and its preference for shaded areas [44]. In the rural village of Kampong Speu, however, *Ae. albolineatus* took the lead over *Ae. albopictus* in terms of relative abundance. This species was only collected in Kampong Speu and only in November. Little is known regarding its biology and behavior. It seems that the coconut husks and small tree holes are the main breeding habitat of this species [45].

*Uranotaenia* was the third most abundant mosquitoe in our study and was also quite diverse, reaching half of *Uranotaenia* species currently recorded in Cambodia [11], which is not surprising since this genus is common in forests. Among them, *Uranotaenia macfarlanei* was the most abundant *Uranotaenia* species. This species was mostly collected in the semi-evergreen forest of Ratanak Kiri during the dry season. According to the literature, this mosquito lay eggs in small pools of dirty water and can be found at about 900 m above sea level [11, 46], but in our study, the adults were collected between 110 and 300 m above sea level. They are known to mainly feed on frogs, and their vectorial status is still unknown [47].

Our *Armigeres* collection was also quite diverse, with 10 of the 26 *Armigeres* species currently present in Cambodia [11]. Interestingly, *Ar. subalbatus* species was present in almost all the sites and was the dominant species in the ranger station in Ratanak Kiri Forest. This species is known to be ecologically flexible and can be commonly found in rural and peri-urban/urban habitats as well [44]. Larvae of this species are found in different container habitats containing nutrient-rich and polluted water, mostly in banana stumps in Cambodia [11].

*Heizmannia* was the fifth most abundant genus in our study, reaching half of the current *Heizmannia* species of Cambodia [11]. Little is known about the biology of *Heizmannia*. Apparently, females of these mosquitoes mainly lay their eggs in tree holes and bamboo, are active during daytime in forests and readily bite humans. *Heizmannia demeilloni* was the most abundant *Heizmannia* species, which was mostly found in in the semi-evergreen forest of Ratanak Kiri and mainly collected during the rainy season. This species is known to breed in bamboo stumps [48].

The mosquitoes belonging to other genera like *Aedeomyia, Anopheles, Coquillettidia, Lutzia, Mansonia, Mimomyia, Tripteroides* and *Toxorhynchites* were scarce in our forests, accounting for only 1.96% (*n* = 184) of our collections. The scarcity of *Anopheles* was particularly surprising given that previous studies have highlighted high diversity and abundance of these mosquitoes in Cambodian forests, including Preah Vihear and Ratanak Kiri [29, 30]. However, during these previous studies, human- and cow-baited traps were chosen, which might be more efficient for *Anopheles* sampling than the type of traps used during our work.

This study also provided predictive relationships between abiotic factors and mosquito abundance for a wide range of species including some uncommon or poorly studied ones. The results clearly demonstrated that when the relative abundance of mosquitoes was positively impacted by altitude it was mainly negatively related to temperature at a species-specific time lag. This could be explained by the fact that temperature generally decreases with altitude [49]. The result of our model combined with the observations from previous studies [49, 50] indicate that lowlands are more suitable for *Ar. subalbatus* occurrence and abundance. Regarding *Ae. albopictus* abundance, the highlands were more suitable, while it was positively impacted by the temperature during the fourth week before collection. This might be explained by the ability of this species to adapt to various ranges of temperature [51].

Additionally, these abiotic factors have been highlighted as important parameters determining the community composition of mosquito species. For instance, the co-occurrence of *Ae. albolineatus, Ar. annulitarsis* and *Cx. bitaeniorhynchus* in the two semi-evergreen forests of Kampong Speu could be explained by the fact that their relative abundance was positively correlated with the altitude and average temperature in the second week prior to the collection. Previous studies demonstrated that mosquito community composition is strongly influenced by landscape [52, 53]. In our case, for logistical reasons, a better characterization of our study site has not been made. This should be undertaken in the future to assess this impact of environmental factors on the mosquito community in these forests.

The forests investigated in this study are located in protected areas of Cambodia. Despite this, many forest-goers rely on timber and non-timber forest products, increasing the deforestation rate, yet to be efficiently regulated in Cambodia. The presence of mosquito species well adapted to living in close vicinity to humans and human settlements indicates the presence of human activities in these areas. The collected mosquitoes that could be indicators of anthropization were *Ae. aegypti* and *Cx. quinquefasciatus*, two domestic mosquito species well adapted to the human environment [42, 54, 55]. The presence of *Anopheles campestris* and *An. baimaii*, whose females are highly antropophilic [56, 57], could also be evidence of human activities in Kampong Speu where these species were only found. Finally, *Cx. gelidus*, which feed on large domestic animals [11, 39], and *Mansonia annulifera*, a highly anthropophilic mosquito biting mainly inside habitations [41], could also be an indicator of anthropization in Siemreap Forest. Surprisingly, despite the human activities observed in Preah Vihear Forest, the mosquito species collected in the three sites were likely either mainly zoophilic or opportunistic. The same finding was observed in Ratanak Kiri Forest.

Our study highlighted a high risk of pathogen emergence/re-emergence in our sites due to the presence of mosquito species of medical importance in these areas. One of the most important features is the abundance of *Cx. pseudovishnui*, a potential vector of JEV [58, 59], which was present in all the sites regardless of the season. Other species collected in this study, *Ae. albopictus, Aedes vexans, Ar. subalbatus, Cx. bitaeniorhynchus, Culex fuscocephala, Cx. gelidus, Cx. quinquefasciatus, Cx. sitiens, Ma. annulifera, Ma. indiana* and *Ma. uniformis*, are also reported to be confirmed or potential vectors of JEV [59–66] and can also transmit other pathogens. For instance, *Ae. albopictus*, the second most abundant mosquito in this work, could transmit several other arboviruses including CHIKV, DENV and ZIKV [67, 68]. This species was also present across the different sites independently of the season. *Armigeres subalbatus*, which was present in almost all sites, is a potential vector of ZIKV [69] and is implicated in the transmission of filaria [70]. *Aedes vexans, Cx. quinquefasciatus* and *Ma. uniformis* could transmit different arboviruses including the Rift Valley fever virus [71]. Also, *Ae. aegypti* is a vector of several pathogens [72] and is considered a major vector of DENV [73]. Finally, seven *Anopheles* species, namely *An. barbirostris, An. campestris, An. karwari, An. maculatus, An. minimus, An. nivipes* and *An. philippinensis*, are reported to be vectors of *Plasmodium* [11, 30]. Furthermore, due to their presence in both forested and rural areas in our study and their zoo-anthropogenic behavior [74–77], *Ae. albopictus, Ar. subalbatus*, *Cx. pseudovishnui* and *Cx. quinquefasciatus* could potentially act as bridge vectors for new emerging pathogens.

The main limitations of the present study are that, for logistical reasons, each site was visited only two times and only two kinds of traps (BG-sentinel and Light trap) were used. Increasing the number of samplings and the diversity of traps in these areas would improve the diversity and density of mosquito fauna.

## Conclusion

This study shows the important diversity of mosquitoes as well as the density of the species of medical importance in four forests in Cambodia which responded differently to meteorological and geographical factors. It also highlights the presence of mosquitoes related to human activities in these supposedly protected areas. Additionally, it emphasizes a high risk of re-emergence of pathogens in these areas due to the abundance of mosquito species that are potentially vectors of pathogens. Finally, the potential emergence of new pathogens in these areas is a public heath consideration due to the presence and abundance of mosquitoes displaying zoo-anthropogenic behavior in forested and rural areas. In fact, these could serve as bridge vectors between sylvatic and anthropogenic pathogens. Further studies using next-generation sequencing methods should therefore be conducted to investigate the pathogen diversity among these mosquitoes, providing information on the risk of disease emergence.

## Supplementary Information

**Additional file 1: Table S1 **Number of mosquitoes collected per forest and per site.

**Additional file 2: Table S2 **Pearson correlation between the relative abundance of mosquito species with relative abundance ≥ 5.

**Additional file 3: Table S3 ***P*-value of the Pearson correlation between the relative abundance of mosquito species with relative abundance ≥ 5.

**Additional file 4: Table S4 **Result of regression model showing the correlation between the presence of species and meteorological/geographical variables. Mosquito species having a relative abundance between 10 and 39 were included in the analysis.

## Data Availability

Data supporting the conclusions of this article are included within the article and its additional files. Raw data are available from the corresponding authors upon reasonable request.
