# Supplementary material for: Mosquito diversity (Diptera: Culicidae) and medical importance in four Cambodian forests
Source: Parasit Vectors. 2023 Mar 21;16:110. doi: 10.1186/s13071-023-05729-w (PMC10029166; doi:10.1186/s13071-023-05729-w)
Supplement: Supplementary file 1 — Additional file 1: Table S1 Number of mosquitoes collected per forest and per site. [file 13071_2023_5729_MOESM1_ESM.docx]

|  |  | **Kampong speu** | | | **Preah Vihear** | | | **Ratanak Kiri** | | | **Siemreap** | | | Total |  |  |  |
| --- | --- | --- | --- | --- | --- | --- | --- | --- | --- | --- | --- | --- | --- | --- | --- | --- | --- |
| **Genus** | **species** | **Site 1** | **Site 2** | **Site 3** | **Site 1** | **Site 2** | **Site 3** | **Site 1** | **Site 2** | **Site 3** | **Site 1** | **Site 2** | **Site 3** | **N** | **% genus** | **% species** | **N sites** |
| *Aedeomyia* | *Ad. catasticta* | 0 | 0 | 0 | 0 | 0 | 0 | 0 | 1 | 0 | 0 | 0 | 0 | 1 | 0.01% | 0.01% | 1 |
| *Aedes* | *Ae. aegypti* | 0 | 0 | 20 | 0 | 0 | 0 | 0 | 0 | 0 | 0 | 0 | 0 | 20 | 42.01% | 0.21% | 1 |
|  | *Ae. albolineatus* | 54 | 68 | 48 | 0 | 0 | 0 | 0 | 0 | 0 | 0 | 0 | 0 | 170 |  | 1.81% | 3 |
|  | *Ae. albopictus* | 332 | 111 | 42 | 131 | 62 | 39 | 97 | 106 | 5 | 166 | 149 | 154 | 1394 |  | 14.84% | 12 |
|  | *Ae. annulirostris* | 0 | 0 | 0 | 2 | 0 | 0 | 0 | 0 | 0 | 0 | 0 | 0 | 2 |  | 0.02% | 1 |
|  | *Ae. caecus* | 0 | 0 | 0 | 0 | 0 | 0 | 1 | 0 | 0 | 0 | 0 | 0 | 1 |  | 0.01% | 1 |
|  | *Ae. desmotes* | 40 | 79 | 8 | 0 | 0 | 0 | 0 | 0 | 0 | 0 | 0 | 0 | 127 |  | 1.35% | 3 |
|  | *Ae. eldridgei* | 42 | 5 | 0 | 0 | 0 | 0 | 0 | 0 | 0 | 0 | 0 | 0 | 47 |  | 0.50% | 2 |
|  | *Ae. elsiae* | 2 | 0 | 0 | 0 | 0 | 0 | 0 | 0 | 0 | 0 | 0 | 0 | 2 |  | 0.02% | 1 |
|  | *Ae. feegradei* | 0 | 0 | 0 | 0 | 0 | 0 | 0 | 3 | 0 | 0 | 0 | 0 | 3 |  | 0.03% | 1 |
|  | *Ae. gardneri imitator* | 5 | 1 | 0 | 52 | 8 | 4 | 37 | 10 | 1 | 166 | 112 | 6 | 402 |  | 4.28% | 11 |
|  | *Ae. ibis* | 1 | 0 | 0 | 2 | 0 | 0 | 1 | 7 | 0 | 0 | 3 | 0 | 14 |  | 0.15% | 5 |
|  | *Ae. imprimens* | 0 | 0 | 0 | 0 | 0 | 0 | 1 | 0 | 0 | 0 | 1 | 0 | 2 |  | 0.02% | 2 |
|  | *Ae. ostentatio* | 0 | 0 | 0 | 0 | 0 | 0 | 4 | 4 | 0 | 0 | 0 | 0 | 8 |  | 0.09% | 2 |
|  | *Ae. prominens* | 1 | 0 | 0 | 0 | 0 | 0 | 0 | 1 | 0 | 1 | 5 | 0 | 8 |  | 0.09% | 4 |
|  | *Ae. thailandensis* | 0 | 0 | 0 | 0 | 0 | 0 | 0 | 1 | 0 | 0 | 0 | 0 | 1 |  | 0.01% | 1 |
|  | *Ae. vexans* | 0 | 0 | 0 | 0 | 2 | 2 | 0 | 0 | 0 | 2 | 2 | 0 | 8 |  | 0.09% | 4 |
|  | *Ae. vittatus* | 2 | 0 | 1 | 0 | 0 | 0 | 0 | 0 | 0 | 0 | 0 | 1 | 4 |  | 0.04% | 3 |
|  | *Ae. sp.* | 381 | 711 | 44 | 91 | 27 | 30 | 59 | 71 | 2 | 59 | 235 | 23 | 1733 |  | 18.45% | 12 |
| *Anopheles* | *An. baimaii* | 2 | 1 | 0 | 0 | 0 | 0 | 0 | 0 | 0 | 0 | 0 | 0 | 3 | 0.70% | 0.03% | 2 |
|  | *An. barbirostris* | 0 | 0 | 0 | 0 | 0 | 0 | 0 | 1 | 0 | 0 | 0 | 0 | 1 |  | 0.01% | 1 |
|  | *An. campestris* | 0 | 0 | 4 | 0 | 0 | 0 | 0 | 0 | 0 | 0 | 0 | 0 | 4 |  | 0.04% | 1 |
|  | *An. interruptus* | 0 | 0 | 0 | 0 | 0 | 0 | 2 | 0 | 0 | 0 | 0 | 0 | 2 |  | 0.02% | 1 |
|  | *An. karwari* | 0 | 0 | 0 | 0 | 0 | 0 | 2 | 0 | 0 | 0 | 0 | 0 | 2 |  | 0.02% | 1 |
|  | *An. maculatus* | 0 | 0 | 0 | 1 | 0 | 0 | 0 | 0 | 0 | 2 | 0 | 1 | 4 |  | 0.04% | 3 |
|  | *An. minimus* | 0 | 0 | 0 | 0 | 0 | 0 | 1 | 0 | 0 | 0 | 0 | 0 | 1 |  | 0.01% | 1 |
|  | *An. nivipes* | 0 | 0 | 2 | 0 | 1 | 0 | 0 | 0 | 0 | 0 | 0 | 0 | 3 |  | 0.03% | 2 |
|  | *An. peditaeniatus* | 0 | 0 | 0 | 0 | 0 | 0 | 0 | 0 | 0 | 1 | 0 | 0 | 1 |  | 0.01% | 1 |
|  | *An. philippinensis* | 0 | 0 | 0 | 0 | 1 | 0 | 0 | 0 | 0 | 0 | 0 | 0 | 1 |  | 0.01% | 1 |
|  | *An. umbrosus* | 0 | 0 | 0 | 0 | 0 | 0 | 1 | 0 | 0 | 0 | 0 | 0 | 1 |  | 0.01% | 1 |
|  | *An. sp.* | 3 | 6 | 9 | 2 | 9 | 2 | 4 | 4 | 0 | 4 | 0 | 0 | 43 |  | 0.46% | 9 |
| *Armigeres* | *Ar. annulitarsis* | 9 | 26 | 2 | 0 | 0 | 0 | 1 | 4 | 0 | 0 | 0 | 0 | 42 | 3.49% | 0.45% | 5 |
|  | *Ar. aureolineatus* | 0 | 0 | 0 | 0 | 0 | 0 | 1 | 0 | 0 | 0 | 0 | 0 | 1 |  | 0.01% | 1 |
|  | *Ar. dolichocephalus* | 0 | 0 | 0 | 0 | 0 | 0 | 2 | 1 | 0 | 0 | 0 | 0 | 3 |  | 0.03% | 2 |
|  | *Ar. flavus* | 1 | 0 | 0 | 0 | 0 | 0 | 0 | 0 | 0 | 0 | 0 | 0 | 1 |  | 0.01% | 1 |
|  | *Ar. kesseli* | 0 | 0 | 0 | 0 | 0 | 0 | 1 | 0 | 0 | 4 | 0 | 4 | 9 |  | 0.10% | 3 |
|  | *Ar. longipalpis* | 0 | 2 | 0 | 0 | 0 | 0 | 0 | 0 | 0 | 0 | 0 | 0 | 2 |  | 0.02% | 1 |
|  | *Ar. malayi* | 2 | 0 | 0 | 0 | 0 | 0 | 0 | 0 | 0 | 0 | 0 | 0 | 2 |  | 0.02% | 1 |
|  | *Ar. omissus* | 2 | 1 | 0 | 0 | 0 | 0 | 0 | 0 | 0 | 0 | 0 | 0 | 3 |  | 0.03% | 2 |
|  | *Ar. subalbatus* | 3 | 6 | 1 | 6 | 2 | 1 | 12 | 75 | 56 | 2 | 0 | 15 | 179 |  | 1.91% | 11 |
|  | *Ar. theobaldi* | 0 | 0 | 1 | 0 | 0 | 0 | 0 | 0 | 0 | 0 | 0 | 0 | 1 |  | 0.01% | 1 |
|  | *Ar. sp.* | 24 | 22 | 2 | 1 | 0 | 1 | 9 | 3 | 16 | 0 | 0 | 7 | 85 |  | 0.91% | 9 |
| *Coquillettidia* | *Cq. crassipes* | 11 | 9 | 7 | 2 | 3 | 0 | 0 | 3 | 0 | 3 | 4 | 0 | 42 | 0.45% | 0.45% | 8 |
| *Culex* | *Cx. bitaeniorhynchus* | 29 | 34 | 7 | 1 | 3 | 1 | 5 | 2 | 0 | 5 | 0 | 0 | 87 | 45.58% | 0.93% | 9 |
|  | *Cx. brevipalpis* | 0 | 0 | 10 | 2 | 5 | 0 | 2 | 0 | 0 | 7 | 1 | 1013 | 1040 |  | 11.07% | 7 |
|  | *Cx. cinctellus* | 105 | 122 | 0 | 0 | 0 | 0 | 0 | 1 | 0 | 2 | 2 | 0 | 232 |  | 2.47% | 5 |
|  | *Cx. fraudatrix* | 0 | 52 | 0 | 0 | 0 | 0 | 1 | 9 | 0 | 0 | 0 | 0 | 62 |  | 0.66% | 3 |
|  | *Cx. fuscocephala* | 4 | 0 | 1 | 0 | 0 | 0 | 0 | 0 | 2 | 1 | 0 | 0 | 8 |  | 0.09% | 4 |
|  | *Cx. gelidus* | 0 | 0 | 0 | 0 | 0 | 0 | 0 | 0 | 0 | 4 | 1 | 1 | 6 |  | 0.06% | 3 |
|  | *Cx. infantulus* | 0 | 0 | 0 | 0 | 0 | 0 | 0 | 0 | 0 | 0 | 0 | 1 | 1 |  | 0.01% | 1 |
|  | *Cx. macdonaldi* | 0 | 0 | 0 | 0 | 0 | 0 | 0 | 0 | 0 | 1 | 1 | 0 | 2 |  | 0.02% | 2 |
|  | *Cx. mimulus* | 6 | 0 | 0 | 0 | 0 | 0 | 2 | 1 | 0 | 1 | 0 | 2 | 12 |  | 0.13% | 5 |
|  | *Cx. nigropunctatus* | 6 | 33 | 3 | 29 | 7 | 0 | 2 | 4 | 0 | 30 | 8 | 4 | 126 |  | 1.34% | 10 |
|  | *Cx. perplexus* | 0 | 0 | 0 | 0 | 0 | 0 | 1 | 0 | 0 | 0 | 1 | 0 | 2 |  | 0.02% | 2 |
|  | *Cx. quinquefasciatus* | 1 | 1 | 19 | 0 | 0 | 0 | 0 | 0 | 0 | 7 | 1 | 0 | 29 |  | 0.31% | 5 |
|  | *Cx. sinensis* | 1 | 1 | 0 | 0 | 0 | 2 | 2 | 0 | 0 | 0 | 0 | 0 | 6 |  | 0.06% | 4 |
|  | *Cx. sitiens* | 3 | 0 | 0 | 0 | 0 | 0 | 3 | 0 | 0 | 0 | 0 | 0 | 6 |  | 0.06% | 2 |
|  | *Cx. pseudovishnui* | 132 | 67 | 13 | 39 | 41 | 17 | 841 | 735 | 17 | 51 | 397 | 8 | 2358 |  | 25.11% | 12 |
|  | *Cx. whitmorei* | 0 | 2 | 0 | 0 | 0 | 0 | 0 | 0 | 0 | 0 | 0 | 0 | 2 |  | 0.02% | 1 |
|  | *Cx. sp.* | 9 | 116 | 5 | 23 | 7 | 3 | 13 | 52 | 3 | 22 | 7 | 42 | 302 |  | 3.22% | 12 |
| *Heizmania* | *Hz. catesi* | 0 | 0 | 0 | 0 | 0 | 0 | 0 | 0 | 0 | 0 | 1 | 0 | 1 | 2.40% | 0.01% | 1 |
|  | *Hz. chengi* | 0 | 0 | 0 | 0 | 0 | 0 | 3 | 0 | 0 | 3 | 0 | 0 | 6 |  | 0.06% | 2 |
|  | *Hz. complex* | 0 | 0 | 0 | 0 | 0 | 0 | 1 | 3 | 0 | 0 | 1 | 0 | 5 |  | 0.05% | 3 |
|  | *Hz. demeilloni* | 0 | 0 | 0 | 4 | 0 | 0 | 8 | 40 | 0 | 0 | 0 | 0 | 52 |  | 0.55% | 3 |
|  | *Hz. reidi* | 0 | 0 | 0 | 0 | 0 | 0 | 1 | 0 | 0 | 0 | 0 | 0 | 1 |  | 0.01% | 1 |
|  | *Hz. sp.* | 31 | 7 | 1 | 15 | 3 | 0 | 17 | 69 | 0 | 2 | 15 | 0 | 160 |  | 1.70% | 9 |
| *Lutzia* | *Lu. vorax* | 0 | 0 | 0 | 0 | 0 | 0 | 0 | 0 | 0 | 1 | 0 | 0 | 1 | 0.03% | 0.01% | 1 |
|  | *Lu. sp.* | 0 | 1 | 0 | 1 | 0 | 0 | 0 | 0 | 0 | 0 | 0 | 0 | 2 |  | 0.02% | 2 |
| *Mansonia* | *Ma. annulifera* | 0 | 0 | 0 | 0 | 0 | 0 | 0 | 0 | 0 | 3 | 0 | 0 | 3 | 0.22% | 0.03% | 1 |
|  | *Ma. indiana* | 0 | 0 | 0 | 0 | 0 | 0 | 1 | 0 | 0 | 4 | 0 | 0 | 5 |  | 0.05% | 2 |
|  | *Ma. uniformis* | 0 | 0 | 2 | 0 | 0 | 0 | 0 | 0 | 0 | 1 | 0 | 7 | 10 |  | 0.11% | 3 |
|  | *Ma. sp.* | 0 | 2 | 0 | 0 | 0 | 0 | 0 | 0 | 0 | 1 | 0 | 0 | 3 |  | 0.03% | 2 |
| *Mimomyia* | *Mi. aurea* | 0 | 1 | 0 | 0 | 0 | 0 | 0 | 0 | 0 | 0 | 0 | 0 | 1 | 0.18% | 0.01% | 1 |
|  | *Mi. hybrida* | 0 | 0 | 0 | 0 | 0 | 0 | 0 | 0 | 0 | 9 | 0 | 0 | 9 |  | 0.10% | 1 |
|  | *Mi. luzonensis* | 0 | 0 | 3 | 0 | 0 | 0 | 0 | 0 | 0 | 0 | 0 | 0 | 3 |  | 0.03% | 1 |
|  | *Mi. sp.* | 1 | 1 | 0 | 1 | 0 | 0 | 0 | 0 | 0 | 1 | 0 | 0 | 4 |  | 0.04% | 4 |
| *Tripteroides* | *Tr. aranoides* | 0 | 2 | 0 | 0 | 0 | 0 | 0 | 0 | 0 | 0 | 0 | 0 | 2 | 0.28% | 0.02% | 1 |
|  | *Tr. caeruleocephalus* | 1 | 0 | 0 | 0 | 0 | 0 | 0 | 0 | 0 | 0 | 0 | 0 | 1 |  | 0.01% | 1 |
|  | *Tr. powelli* | 0 | 0 | 0 | 0 | 0 | 0 | 0 | 0 | 0 | 0 | 2 | 0 | 2 |  | 0.02% | 1 |
|  | *Tr. sp.* | 3 | 14 | 0 | 4 | 0 | 0 | 0 | 0 | 0 | 0 | 0 | 0 | 21 |  | 0.22% | 3 |
| *Toxorhynchites* | *Tx. sp.lendens* | 0 | 0 | 0 | 4 | 1 | 0 | 0 | 0 | 0 | 0 | 0 | 0 | 5 | 0.09% | 0.05% | 2 |
|  | *Tx. sp.* | 0 | 0 | 0 | 1 | 0 | 0 | 0 | 1 | 0 | 0 | 1 | 0 | 3 |  | 0.03% | 3 |
| *Uranotaenia* | *Ur. bicolor* | 0 | 0 | 0 | 0 | 0 | 0 | 0 | 5 | 0 | 1 | 0 | 0 | 6 | 4.45% | 0.06% | 2 |
|  | *Ur. bimaculiala* | 0 | 0 | 0 | 0 | 0 | 0 | 0 | 0 | 0 | 5 | 0 | 0 | 5 |  | 0.05% | 1 |
|  | *Ur. campestris* | 0 | 0 | 0 | 0 | 0 | 0 | 0 | 0 | 0 | 1 | 0 | 0 | 1 |  | 0.01% | 1 |
|  | *Ur. koli* | 0 | 18 | 0 | 0 | 0 | 0 | 0 | 1 | 0 | 0 | 0 | 0 | 19 |  | 0.20% | 2 |
|  | *Ur. longirostris* | 0 | 4 | 0 | 0 | 0 | 0 | 0 | 0 | 0 | 18 | 0 | 0 | 22 |  | 0.23% | 2 |
|  | *Ur. lutescens* | 0 | 3 | 0 | 0 | 0 | 0 | 0 | 0 | 0 | 0 | 0 | 0 | 3 |  | 0.03% | 1 |
|  | *Ur. macfarlanei* | 0 | 13 | 4 | 0 | 0 | 0 | 2 | 64 | 0 | 0 | 0 | 0 | 83 |  | 0.88% | 4 |
|  | *Ur. maxima* | 0 | 0 | 0 | 0 | 0 | 0 | 0 | 1 | 0 | 0 | 0 | 0 | 1 |  | 0.01% | 1 |
|  | *Ur. metatarsata* | 1 | 0 | 0 | 0 | 0 | 0 | 0 | 0 | 0 | 5 | 0 | 0 | 6 |  | 0.06% | 2 |
|  | *Ur. micans* | 0 | 3 | 0 | 0 | 0 | 0 | 0 | 0 | 0 | 0 | 0 | 0 | 3 |  | 0.03% | 1 |
|  | *Ur. rampae* | 0 | 1 | 0 | 0 | 0 | 0 | 1 | 0 | 0 | 0 | 0 | 0 | 2 |  | 0.02% | 2 |
|  | *Ur. testacea* | 0 | 5 | 0 | 0 | 0 | 0 | 0 | 0 | 0 | 0 | 0 | 0 | 5 |  | 0.05% | 1 |
|  | *Ur. trilineata* | 0 | 0 | 0 | 0 | 0 | 0 | 0 | 0 | 0 | 1 | 0 | 0 | 1 |  | 0.01% | 1 |
|  | *Ur. sp.* | 12 | 56 | 1 | 9 | 1 | 0 | 36 | 105 | 21 | 18 | 2 | 0 | 261 |  | 2.78% | 10 |
|  | Unidentified | 1 | 0 | 0 | 1 | 0 | 0 | 1 | 4 | 0 | 1 | 2 | 0 | 10 |  |  |  |
|  | **Total** | **1263** | **1607** | **260** | **424** | **183** | **102** | **1179** | **1392** | **123** | **616** | **954** | **1289** | **9392** |  |  |  |
|  | **Number of genera** | **9** | **11** | **9** | **11** | **7** | **4** | **7** | **10** | **4** | **10** | **7** | **5** | **13** |  |  |  |
|  | **Number of species** | **28** | **31** | **21** | **16** | **14** | **8** | **31** | **26** | **6** | **31** | **20** | **13** | **85** |  |  |  |
|  | **Shannon index (H')** | **1.95** | **2.49** | **2.36** | **1.57** | **1.56** | **1.17** | **0.87** | **1.29** | **0.9** | **1.99** | **1.21** | **0.63** |  |  |  |  |
|  | **Simpson index (D)** | **0.23** | **0.11** | **0.14** | **0.3** | **0.31** | **0.42** | **0.66** | **0.48** | **0.53** | **0.23** | **0.4** | **0.71** |  |  |  |  |
|  | **Pielou's evenness (J)** | **0.23** | **0.21** | **0.27** | **0.26** | **0.28** | **0.31** | **0.13** | **0.20** | **0.33** | **0.23** | **0.23** | **0.13** |  |  |  |  |
