# Supplementary material for: Mosquito diversity (Diptera: Culicidae) and medical importance in four Cambodian forests
Source: Parasit Vectors. 2023 Mar 21;16:110. doi: 10.1186/s13071-023-05729-w (PMC10029166; doi:10.1186/s13071-023-05729-w)
Supplement: Supplementary file 4 — Additional file 4: Table S4 Result of regression model showing the correlation between the presence of species and meteorological/geographical variables. Mosquito species having a relative abundance between 10 and 39 were included in the analysis. [file 13071_2023_5729_MOESM4_ESM.docx]

| **Factors** | **Species** | **IRR** | **IC_95_ inf** | **IC_95_ sup** | **p-value** |
| --- | --- | --- | --- | --- | --- |
| Altitude | *Ae. aegypti* | 0.99 | 0.98 | 1.00 | 0.12 |
|  | *Ae. ibis* | 1.00 | 0.99 | 1.01 | 0.76 |
|  | *Cx. mimulus* | 1.00 | 1.00 | 1.01 | 0.63 |
|  | *Cx. quinquefasciatus* | 1.00 | 0.99 | 1.00 | 0.66 |
|  | *Ma. uniformis* ^(-)^ | 0.99 | 0.96 | 1.00 | 0.01 |
|  | *Ur. koli* ^(+)^ | 1.01 | 1.00 | 1.02 | 2 x 10^-4^ |
|  | *Ur. longirostris* | 1.00 | 0.99 | 1.01 | 0.29 |
| Precipitation 1^st^ week before the collection | *Cx. mimulus* | 0.84 | 0.63 | 1.00 | 0.05 |
|  | *Ur. longirostris* ^(-)^ | 0.75 | 0.49 | 0.98 | 0.02 |
| Precipitation 2^nd^ week before the collection | *Ur. koli* ^(+)^ | 1.19 | 1.05 | 1.40 | 0.01 |
| Precipitation 3^rd^ week before the collection | *Ae. ibis* ^(+)^ | 1.07 | 1.01 | 1.13 | 0.02 |
|  | *Cx. quinquefasciatus* | 0.94 | 0.82 | 1.02 | 0.13 |
| Precipitation 4^rd^ week before the collection | *Ae. aegypti* ^(-)^ | 0.78 | 0.54 | 0.95 | 0.01 |
|  | *Ma. uniformis* | 0.92 | 0.77 | 1.05 | 0.22 |
| Temperature 1st week before collection | *Ae. ibis* | 0.92 | 0.66 | 1.24 | 0.54 |
|  | *Ur. koli* | 0.74 | 0.43 | 1.23 | 0.05 |
| Temperature 2^nd^ week before collection | *Ae. aegypti* ^(+)^ | 1.30 | 0.97 | 1.84 | 0.01 |
| Temperature 4^th^ week before collection | *Cx. mimulus* | 1.19 | 0.85 | 1.74 | 0.32 |
|  | *Cx. quinquefasciatus* | 1.27 | 0.94 | 1.83 | 0.06 |
|  | *Ma. uniformis* ^(+)^ | 1.91 | 1.10 | 4.29 | 0.01 |
|  | *Ur. longirostris* ^(+)^ | 2.53 | 1.50 | 5.74 | 7 x 10^-6^ |

*Abbreviation:* IRR, Incidence Ratio Rate. ANOVA test. (+): positive correlation, (-): negative correlation.
